# Ecological Distribution of Virulent Multidrug-Resistant *Staphylococcus aureus* in Livestock, Environment, and Dairy Products

**DOI:** 10.3390/antibiotics11111651

**Published:** 2022-11-18

**Authors:** Basma Badawy, Mahmoud Elafify, Alshimaa M. M. Farag, Samar M. Moustafa, Mohamed Z. Sayed-Ahmed, Amira A. Moawad, Abdelazeem M. Algammal, Hazem Ramadan, Mahmoud Eltholth

**Affiliations:** 1 Department of Hygiene and Zoonoses, Faculty of Veterinary Medicine, Mansoura University, Mansoura 35516, Egypt; 2 Department of Food Hygiene and Control, Faculty of Veterinary Medicine, Mansoura University, Mansoura 35516, Egypt; 3 Department of Internal Medicine, Infectious and Fish Diseases, Faculty of Veterinary Medicine, Mansoura University, Mansoura 35516, Egypt; 4 Department of Zoonoses, Faculty of Veterinary Medicine, Benha University, Benha 13511, Egypt; 5 Department of Pharmacy Practice, College of Pharmacy, Jazan University, Jazan 82722, Saudi Arabia; 6 Friedrich-Loeffler-Institut Institute of Bacterial Infections and Zoonoses, Naumburger Str. 96a, 07743 Jena, Germany; 7 Animal Health Research Institute, Agriculture Research Center (ARC), Giza 12618, Egypt; 8 Department of Bacteriology, Immunology and Mycology, Faculty of Veterinary Medicine, Suez Canal University, Ismailia 41522, Egypt; 9 Global Academy of Agriculture and Food Systems, The Royal (Dick) School of Veterinary Studies, University of Edinburgh, Easter Bush Campus, Midlothian, Edinburgh EH25 9RG, UK; 10 Department of Animal Medicine, Faculty of Veterinary Medicine, Kafrelsheikh University, Kafrelsheikh 33516, Egypt; 11 Department of Health Studies, Royal Holloway University of London, Egham TW20 0EX, UK

**Keywords:** *Staphylococcus aureus*, cattle, buffaloes, ecology, antimicrobial resistance, methicillin-resistant, enterotoxin, one health

## Abstract

*Staphylococcus aureus* is one of the most common causes of mastitis, leading to severe economic losses in the dairy industry. It is also zoonotic, with potential risks to public health. This study aimed to detect the occurrence of *S. aureus*-resistant strains isolated from cattle, buffalo, their environment, milk and dairy products; and to investigate the extent of animal, ecological, and food contamination by methicillin-resistant (MRSA) or enterotoxigenic *S. aureus*. Samples (*n* = 350) were collected from four animal (two cattle and two buffalo) farms, i.e., their environment. Thirty Karish cheese samples were collected from 10 markets in Mansoura, Egypt. *S. aureus* was detected in 17.9%, 17.6%, and 16.7% of samples collected from cattle, buffalo and Karish cheese, respectively. About 19% of isolated *S. aureus* strains carried the *mec*A gene. The distribution of the *mec*A gene was high in isolates from Karish cheese (60%), followed by samples collected from buffalo (16.2%) and cattle (16%). More than 34% of isolated *S. aureus* strains were enterotoxigenic, and the presence of enterotoxin genes was higher in isolates from Karish cheese (80%) than those from cattle (48%) and buffalo (18.9%). The most predominant enterotoxin gene among isolated *S. aureus* strains was the *sea* gene (26.9%), followed by *sec* (4.5%) and *sed* (3%) genes. Isolated strains were resistant to clindamycin (100%), kanamycin (97%), nalidixic acid (86.6%), cefotaxime (73.1%) sulphamethazole—trimethoprim (65.6%). Meanwhile, 95.5%, 94%, 86.6% and 77.7% of *S. aureus* strains were sensitive to ciprofloxacin, amikacin, imipenem and both cefoxitin and gentamycin, respectively. In conclusion, the presence of enterotoxigenic- and methicillin-resistant *S. aureus* strains in animals, their environment, and dairy products represents a public health concern, particularly in small-scale dairy farms in Egypt. To reduce the risk of infection of livestock and humans with resistant strains, strict regulations and guidelines for antimicrobial use in such a system are urgently required.

## 1. Introduction

*Staphylococcus aureus* (*S. aureus*) is an opportunistic pathogen that is considered to be one of the leading causes of mastitis in dairy cattle [1,2,3,4]. A significant proportion (49.7%) of subclinical bovine mastitis was caused by *Staphylococc*i, of which *S. aureus* was the predominant species (64.95%) [5]. Mammary gland infection with *Staphylococcus aureus* remains a main worldwide problem to the dairy industry due to its contagiousness, pathogenicity, persistence in the cow environment, colonization of skin or mucosal epithelia, and the poor therapeutic efficacy of different applied antimicrobials [6]. Globally, mastitis is one of the most common diseases affecting dairy herds, leading to significant economic losses due to the increased somatic cell count (SCC), physical changes in milk, decreased milk production, increased costs of veterinary drugs, and early culling of infected animals [7]. Most *S. aureus* strains (94%) are resistant to penicillin and its derivatives due to the production of the penicillinase enzyme [8,9]. Strains of *S. aureus* that are resistant to methicillin are known as methicillin-resistant *Staphylococcus aureus* (MRSA) [10]. The latter could be determined phenotypically by antimicrobial susceptibility testing to cefoxitin and/or oxacillin and genetically by the detection of the *mec*A gene using PCR. The *mec*A gene mainly encodes for a modified penicillin-binding protein (PBP2a) that leads to antimicrobial resistance (AMR) [11]. *S. aureus* possesses different virulence factors, such as extracellular toxins, of which the enterotoxins cause food poisoning and others are responsible for causing many clinical manifestations in humans and animals [11]. Enterotoxins are thermostable and cause food poisoning in humans when contaminated foods are consumed [11,12]. *S. aureus* and its enterotoxins are the third highest cause of foodborne illnesses globally [2,13]. It can colonize and persist in different environments, inanimate objects, or media [14]. Virulence factors that have an important role in pathogenicity are adhesins and surface proteins, such as proteins A, *Staphylococcal* enterotoxins (SEs), and β-Hemolysin (Hlb) [15]. The organism has numerous ways to overcome phagocytosis and invade the udder, causing chronic inflammation [16].

The presence of *S. aureus* in milk indicates low hygienic standards adopted during the milking process [17]. Dairy farms are major reservoirs of *S. aureus*; it can be transmitted from cattle, farm environment, and farm workers to bulk tank milk (BTM), causing a public health hazard [2]. 

The resistance of *S. aureus* to antimicrobial agents is a growing challenge and can hinder the treatment of infections [1]. In Egypt, a high proportion of *S. aureus* isolates from humans and bovines were resistant to antibiotics [18]. Unfortunately, the use of antimicrobials in livestock production is poorly regulated in Egypt [19]. This resistance might be due to the fact that antimicrobials such as tetracycline, quinolones, and beta lactams are still used as growth promotors and feed additives for animals [20,21]. However, Egypt has launched a five-year National Action Plan (NAP) on AMR (2017–2022), aiming for the consistent investigation of AMR and promoting the judicious use of antimicrobials for humans and animals [21].

In Egypt, the prevalence and genotypes of circulating *S. aureus* and other *Staphylococci* among dairy cattle and buffaloes have been reported in several studies [22,23], yet scarce and very limited data about the occurrence of antimicrobial-resistant strains of *S. aureus* in animals and their environment still exist. This study was carried out to detect the occurrence of antimicrobial-resistant *S. aureus* strains isolated from cattle, buffalo, their environment, and milk and dairy products and investigate the extent of animal, ecological, and food contamination by MRSA or enterotoxigenic *S. aureus*.

## 2. Results 

### 2.1. Occurrence of Staphylococcus aureus in Examined Samples

Generally, there was no significant difference between the occurrence of *S. aureus* in samples from different sources (Table 1). The occurrence of *S. aureus* from cattle environments (15%) was insignificantly (*p* = 0.640) lower than buffaloes (17.5%). There was no significant difference in the level of contamination of milk and feed samples by *S. aureus* (*p* = 0.470 and *p* = 0.058, respectively) either in cattle or buffalo farms. *S. aureus* was isolated from 16.7% of Karish cheese samples.

### 2.2. Occurrence and Distribution of Staphylococcus aureus at the Farm Level

Results (Table 2) showed that there is no significant difference between different types of samples among cattle farms. *S. aureus* was isolated from 17.9% of samples collected from cattle farms (14.3% and 21.4% from farms I and II, respectively). On buffalo farms, it was isolated from 17.6% (5.7% and 29.5% from farms III and IV, respectively). There was a statistically significant difference (*p* = 0.027) in animal samples collected from buffalo farms III and IV (8.9%, and 26.8%, respectively). Milk samples from buffalo farm IV were contaminated with *S. aureus* (40%, *p* = 0.003). Environmental contamination by *S. aureus* was significantly (*p* ≤ 0.001) higher in buffalo farm IV (31.7%) than in farm III (3.3%). Feed contamination was recorded only in farms II and IV (20% and 66.7%, respectively). There was a significant difference (*p* = 0.001) in the presence of *S. aureus* between buffalo farms III (5.7%) and IV (29.5%). There was no significant difference (*p* = 0.06) in the level of contamination of water samples collected from cattle farms.

### 2.3. Occurrence and Distribution of MRSA and Enterotoxigenic Staphylococcus aureus Isolated from Different Sources

More than 19% of isolated *S. aureus* strains were *mec*A gene positive, of which 60%, 16.2%, and 16% of the gene were from Karish cheese, buffalo and cattle samples, respectively). Around 34% of isolated *S. aureus* strains were enterotoxigenic; enterotoxin genes were detected in samples from Karish cheese (80%), cattle (48%), and buffalo (18.9%). The most predominant enterotoxin gene among isolated *S. aureus* strains was the *sea* gene (26.9%) followed by *sec* (4.5%) and *sed* (3%) genes. The enterotoxin *seb* gene was not detected in this study (Table 3).

### 2.4. Antimicrobial Resistence and Multiple Antimicrobial Resistance (MAR) Index of Isolated Staphylococcus aureus Strains

The results for antimicrobial resistance (Table 4) showed that 86.6% of *S. aureus* exhibited multidrug resistance (MDR). All isolates were found to be resistant to clindamycin. Ninety-seven percent, 86.6%, 73.1%, and 65.7% of *S. aureus* isolates were resistant to kanamycin, nalidixic acid, cefotaxime and sulphamethoxazol-trimethoprim, respectively. On the other hand, 95.5% and 94% of the recovered strains were sensitive to ciprofloxacin and amikacin, respectively, followed by imipenem (86.6%), cefoxitin and gentamycin (77.7% each).

The MAR index showed a significant difference (*p* = 0.024) between samples collected from cattle and buffalo (0.519 ± 0.235 and 0.382 ± 0.225, respectively). Also, there was a significant difference (*p* = 0.035) between samples from livestock and dairy products (0.519 ± 0.260) compared to samples from the environment (0.394 ± 0.211) (Table 5).

The MAR index was calculated by dividing the total number of antimicrobial resistances for each isolate by the total number of antimicrobials tested [21]. The results of the MAR index for different samples are shown in Table 6. The mean MAR index (0.70 ± 0.216) for samples from Karish cheese was the highest, followed by milk samples (0.621 ± 0.161), bedding materials (0.499 ± 0.174), and rectal swabs (0.452 ± 0.319). On the other hand, the MAR index for boot swabs was the lowest (0.321 ± 0.100). 

**Figure 1:**
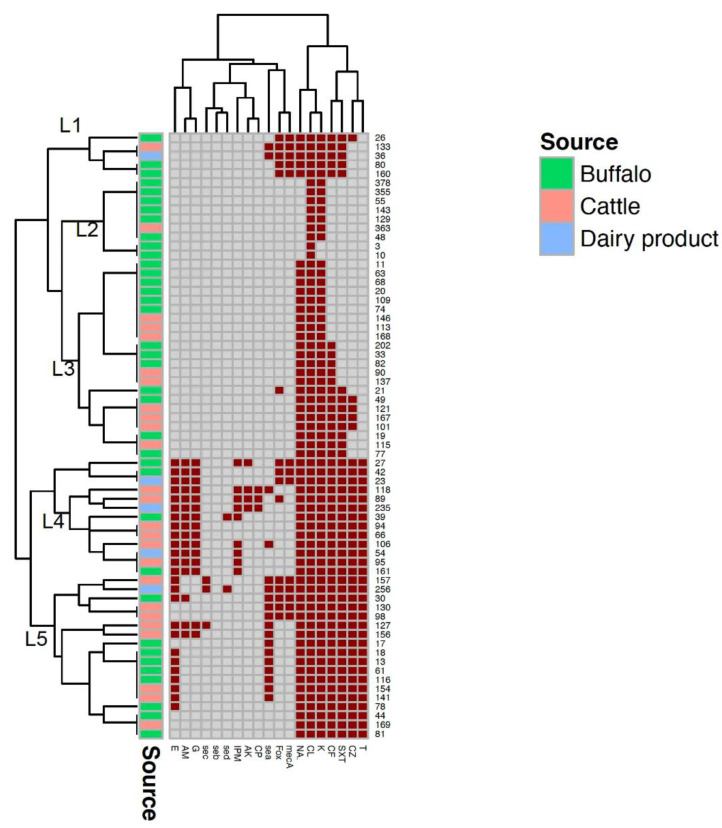
A heatmap showing the distribution of the antimicrobial resistance phenotypes, *mec*A and enterotoxin genes among the examined *Staphylococcus aureus* isolates from cattle, buffalo, and dairy products. Dark red squares indicate phenotypic resistance, the presence of *mec*A and enterotoxin genes; grey squares indicate phenotypic susceptibility and absent genes. Five clusters (L1–L5) are indicated in the figure.

Based on Heatmap and hierarchical clustering, isolates were classified into five clusters (L1, L2, L3, L4 and L5) according to the distribution of AMR phenotypes, *mec*A and enterotoxin genes (Figure 1). Clusters L1, L4 and L5 contained isolates from samples collected from cattle, buffalo, and dairy products, whereas L2 and L3 were from isolates obtained from cattle and buffalo. Although there was no specific trend for clustering isolates from different sources, some isolates from different sources displayed identical profiles of AMR phenotypes, *mec*A and enterotoxin genes. For instance, isolates 133 and 36 recovered from cattle and dairy products had the same AMR phenotypes, and both carried *mec*A and *sea* genes.

## 3. Discussion

*S. aureus* is one of the major causes of mastitis in dairy cattle, resulting in severe health issues and economic losses. Molecular and epidemiological studies of *S. aureus* from animals, environment, and dairy products in Egypt are scarce. To our knowledge, this is the first study to explore the ecology and the co-circulation of multidrug-resistant *S. aureus* in livestock, dairy products and the environment. 

Our results showed a lower percentage of *S. aureus* from cattle and buffalo than in a previous study in Egypt, in which *S. aureus* was detected in 50% to 83% of samples from dairy cattle and buffalo commercial farms and smallholders [24]. In another study, the organism was detected in 72.5% of animals, barn environments, milking equipment, bulk milk tank (BMT), and workers [25]. In a third study, it was detected in 36.3% and 31% of milk samples collected from healthy cattle and buffalo, respectively [26]. Our results were higher than El-Ashker et al., who detected *S. aureus* in 5.6% of milk samples collected from both small dairy householders and well-organized farms [22]. Moreover, the organism was detected in 21.1% of milk samples collected from 140 household dairy cattle and buffalo with mastitis [27]. Variations in *S. aureus* occurrence may be due to the differences in the type of samples, the health status of the animals, the level of hygiene and the season of sampling [28]. A high prevalence of *S. aureus* may be due to the ability of the organism to survive in the udder, and under certain circumstances, it becomes capable of inducing chronic and subclinical infections and acting as a source of infection for other healthy cattle [29]. 

Our detection rate of *S. aureus* from feces and udder swabs from cattle was lower than [25], who detected it in 55.6% of samples. This may be due to the fact that samples in the latter study were collected from open yards with cow sheds, earth floor system and a parlor that depended on pipeline milking machines. The degree of udder skin contamination may be attributed to the persistent association between udder skin and intra-mammary infection (IMI) in dairy cows [30], and there was a minor role of teat skin contamination in IMI of *S. aureus* previously reported [31]. This indicates that there is an association between teat skin colonization and IMI with *S. aureus.* It was concluded that *S. aureus* on teat skin might be a risk factor for IMI. Therefore, proper teat skin hygiene and sanitation are recommended before and after the milking process.

In this study, the level of contamination of buffalo milk contamination by *S. aureus* was higher than in cattle milk; this was similar to a previous study conducted by Abo-Shama et al. [32], in which the organism was detected in buffalo and cattle milk at 46% and 37%, respectively. However, our results were higher than other studies, in which 42%, 36%, and 31% of milk samples from animals with mastitis, cattle with sub-mastitis and buffalo were positive, respectively [23,26]. Our results were also different to studies conducted in Turkey (56%) [33], China (46% and 43%) [34,35] and Ethiopia (15%) [29]. These variations might be attributed to the geographical area, climate conditions, animal species, farm hygiene, the health status of the animals, milking methods and hygiene [36].

Limited data are available on the prevalence of *S. aureus* in animal environments worldwide [2]. There is a need for further epidemiological and ecological studies to better explain the transmission of the pathogenic *S. aureus* between livestock, the environment, food chains and humans [37]. In this study, the level of *S. aureus* detection from environmental samples was in agreement with [28] and [2,38,39], who concluded that farm environments act as a vehicle for *S. aureus* transmission to the farm equipment, cattle, workers and BMT.

*S. aureus* was detected in Karish cheese at a lower rate than [40], in which 73.3% of Karish cheese samples processed from raw milk were positive. However, it was higher than [41] in which 5% and 13% of samples from supermarkets and street vendors were contaminated, respectively. The frequent isolation of *S. aureus* from raw milk intended for cheese production without pasteurization, dairy processing equipment, and environments, as well as food handlers, indicated its introduction to the dairy product supply chain [42]. The critical points of contamination of raw milk with *S. aureus* are the milking process, manure, milking collection facilities and handling during transportation [43]. Therefore, hygienic standards during the production, transportation, and sale of raw milk and dairy products should be carefully monitored.

Resistance to methicillin is attributed to the existence of the *mec*A and/or *mec*C gene(s) on the *S. aureus* chromosomes. Methicillin is stable in the presence of *β-lactamase* enzymes and is effective in the treatment of *S. aureus* infections, but not MRSA [44]. The percentage of *mec*A was lower than in several studies conducted in Egypt [11,22,23,26,45]. This may be due to the differences in the health status of animals. The detection of MRSA was higher than [2]. In our study, few isolates were resistant to cefoxitin and tested negative for *mec*A. This was in agreement with [46] and may be attributed to the existence of the *mecC* gene.

The results of enterotoxigenic *S. aureus* strains were similar to [26]. The percentages of detection of enterotoxin genes, *sea*, *sec*, *sed*, and *seb* were in agreement with [26], who detected the *sea*, and *sec* genes in 27%, and 7% of samples, respectively, while the *seb* and *sed* genes were not detected. The gene coding for enterotoxin A, *sea*, was the most frequently found, as in studies by [11,47]. This toxin is thermostable, not inactivated by pasteurization, and maintains some biological activity after 28 min at 121 °C [48]. The majority of *S. aureus* infections and outbreaks, attributed to the consumption of contaminated milk or milk powder, were associated with *sea, sed*, *sec* and *seb* toxins [48,49]. High percentages of *sea*, *sec*, *sed* and *seb* enterotoxin genes were detected in raw and pasteurized milk [47]. Also, a higher detection rate of *S. aureus* enterotoxins from animals, food and humans was reported by [50]. The sources of enterotoxin and *mec*A genes in the tested *S. aureus* strains were animal, environment, milk and Karish cheese. These pose a potential risk of transmission of antimicrobial resistance to human consumers.

The percentage of MDR *S. aureus* in this study was similar to a previous study in Egypt [23], where 83.3% of *S. aureus* isolates were MDR, with a high resistance against ampicillin (95.2%), penicillin (83.3%) and lower resistance against gentamicin (23.8%), amikacin (16.7%) and ciprofloxacin (14.3%) [23]. MDR of our *S. aureus* strains was high compared to other research studies worldwide. Other studies from Egypt reported a lower MDR percentage than the present study, in which 52.4% [27] and 38.2% [11] of *S. aureus* strains were MDR *S. aureus* strains. The phenotypic MDR phenomenon is mainly attributed to the frequent use of antimicrobials as well as the encoding of some antibiotic-resistant genes. The result of antibiogram agreed with [51], who found that *S. aureus* isolated from milk were sensitive to ciprofloxacin. In contrast, all *S. aureus* isolates were susceptible to sulphamethazole—trimethoprim, erythromycin, gentamicin, ciprofloxacin, followed by penicillin (88.89%) and tetracycline (61.11%) [29]. *S. aureus* strains from milk samples were found to be resistant to penicillin, tetracycline and cefoxitin, with a prevalence rate of 64.3%, 59.5%, and 35.7%, respectively.

The presence of high percentages of MAR *S. aureus* from dairy cattle has been reported worldwide, 100% MAR from Egypt [11], 98.3%, 50%, and 62% from Ethiopia, Italy, and South Africa, respectively [52]. The difference in the MAR index may be attributable to the difference in the prevalence of antimicrobial-resistant genes that are responsible for MDR [53]. The current farming practices make farm animals vulnerable to the development and acquisition of new resistance mechanisms that can propagate into the community and cause a significant risk to the human population [54].

In conclusion, *S. aureus* is an important veterinary and public health issue, particularly for the small-scale dairy herds in Egypt. Further investigations into the prevalence, circulation and AMR of *S. aureus* and other pathogens with public health importance are required. Strict regulations for antimicrobial use in livestock production are highly recommended.

## 4. Materials and Methods

### 4.1. Study Design and Samples Collection

For this study, four dairy farms (2 cattle and 2 buffalo) and 10 markets were purposively selected from the Mansoura district, Dakahlia Governorate, the Eastern Nile Delta region of Egypt. These farms have a history of a sharp decrease in milk production, and the milking process is manual with an absence of veterinary supervision. The frequently used antimicrobials were penicillin, amoxycillin, tetracycline tylosin, and enrofloxacin. The markets are small local markets in rural and peri-urban areas where farmers sell dairy products and other goods. 

In total, 350 samples were collected aseptically from the four farms and their environment, and 30 Karish cheese (made from raw buffalo milk) were collected from 10 markets, three each. Samples from animals included rectal swabs, milk, and udder swabs, 50 each. Samples from farm environments included water, feed, bedding materials, and boot swabs from personnel, 50 each (Table 7). Samples were collected and transported in an icebox to the Animal, Poultry and Environmental Hygiene Laboratory, Faculty of Veterinary Medicine, Mansoura University, Egypt within 2 h of collection. The collection and processing of samples were according to [25,28].

### 4.2. Microbiological and Molecular Characterization of S. aureus

- Isolation and identification of *S. aureus*

All samples were pre-enriched in tryptone soy broth (TSB, Oxoid, Basingstoke, UK) overnight, then cultured on Baired Parker Agar (Oxoid, UK) supplemented with Egg Yolk Tellurite (50 mL/L) (Oxoid, UK). Cultured plates were incubated at 37 °C for 18–24 h, followed by subculture on mannitol salt agar for confirmation. Morphological and biochemical identifications were conducted according to [25] and the thermostable nuclease test “D-Nase activity” [39].

- Molecular characterization

DNA was extracted from the purified strains using a GeneJET Genomic DNA Purification Kit (Fermantas and USA) according to the manufacturer’s instructions. Conventional PCR was performed to determine the *nuc* gene specific for *S. aureus* species, the *mec*A gene for MRSA, *sea*, *seb*, *sec* and *sed* for enterotoxin detection. The primer sequences used for all tested genes and their amplicon sizes were illustrated (Table 8).

- Amplification of *nuc*, enterotoxin and *mec*A genes

A multiplex PCR for the identification of *nuc* and *mec*A genes was performed as for the method described by [57]. Multiplex PCR for enterotoxin genes was done according to the method performed by [47].

### 4.3. Phenotypic antimicrobial Resistance of Isolated S. aureus (Antibiogram)

AMR was performed by the agar disk diffusion method on Mueller–Hinton agar (Difco, Franklin Lakes, NJ, USA), as recommended by the Clinical and Laboratory Standards Institute, CLSI, 2020 [58]. Frequently applied antimicrobials for *S. aureus* infection in humans and animals were selected to be tested against the isolated strains of *S. aureus*. About 14 antimicrobial discs (Oxoid, UK) related to different classes of antimicrobials were used (Table 4). The bacterial culture was uniformly spread on the surface of the agar. Then the antimicrobials discs were placed over the surface of the inoculated plate. Moreover, the plate was then incubated at 35 °C for 24 h and checked for the growth of the bacterium around the antibiotic discs. Examined strains were assessed as susceptible, intermediate, or resistant in accordance with breakpoints supplied by the CLSI (2020) guidelines for *S. aureus* ATCC 25,923. To ensure data compatibility, the experiment was repeated with positive and negative controls, respectively. The positive control (quality control organism) was *S. aureus* ATCC 25,923. For the negative control, 30 μL of sterile distilled water was pipetted onto a blank disc (typically 6 mm in diameter). The MAR index was calculated by dividing the total number of antimicrobial resistances for each isolate by the total number of antimicrobials tested. A MAR index value greater than 0.2 means the isolates originated from a high-risk source of contamination where antimicrobials are massively applied [21,59]. Strains exhibiting resistance to at least one antimicrobial drug in three or more antimicrobial classes were considered to be MDR strains [21].

### 4.4. Statistical Analyses

Data were analyzed using the Statistical Package for Social Sciences (IBM SPSS Statistics for Windows, Version 26.0. Armonk, NY, USA: IBM Corp). The normality of data was first tested with a one-sample Kolmogorov-Smirnov test. The smaller the *p* value obtained, the more significant the results. The Fisher exact test was used when the expected cell count was less than 5. In the present study, the occurrence of *S. aureus,* either enterotoxigenic or MRSA, in two different species of animals (cattle and buffalo), their environmental contamination, milk and milk products were compared by using chi-square and Fisher exact tests for comparison of two or more groups of categorical variables. Regarding the MAR index differentiation between cattle, buffalo, their environment and milk products, the mean and SD (standard deviation) were used to identify which of these categories (animal species, environmental, milk products) had a high risk of MAR. The mean and *p* value of the MAR index for samples from different sources were calculated for comparing samples from rectal, udder, boot swabs, milk, Karish cheese, water and feed. In order to cluster the isolates from different sources according to their AMR phenotypes, the presence of *mec*A and enterotoxin genes, a heatmap with hierarchical clustering was generated using the R packages “Heatmap” and “RColorBrewer” as previously described [11]. The presence of a gene or a resistance phenotype scored 1, whereas the absence of a gene or a susceptible phenotype scored 0. The binary data (0/1) were then imported into R software (version 3.6.1; https://www.r-project.org, accessed on 17 September 2021).

## 5. Conclusions

Results from this study revealed a high level of contamination in the animal environment, milk and dairy products with MDR *S. aureus* strains that impose a public health hazard. These findings may be attributable to a lack of hygiene and veterinary supervision and, consequently, improper use of antimicrobials in the small-scale dairy sector in Egypt. Strict regulations and guidelines for antimicrobial use in such systems are urgently required to reduce the risk of infection of livestock and humans with *S. aureus*-resistant strains. Further examination of these isolates using whole-genome sequencing will be helpful to provide insights into the genomic relatedness of isolates from different sources.

## Figures and Tables

**Table 1:** Occurrence of *S. aureus* in samples collected from cattle, buffalo, milk and their environments.

Type of Sample	Cattle		Buffalo		*p* Value
	*n*	Positive *n* (%)	No. Examined	Positive *n* (%)	
Rectal swabs	20	5 (25.0)	30	4 (13.3)	0.292
Udder swabs	20	5 (25.0)	30	5 (16.7)	0.470
Milk	20	3 (15.0)	30	7 (23.3)	0.470
subtotal	60	13 (21.7)	90	16 (17.8)	0.554
Bedding material	20	4 (20.0)	30	4 (13.3)	0.528
Boot swabs	20	3 (15.0)	30	5 (16.7)	0.874
Feed	20	2 (10.0)	30	10 (33.3)	0.058
Water	20	3 (15.0)	30	2 (6.7)	0.335
subtotal	80	12 (15.0)	120	21 (17.5)	0.640
Total	140	25 (17.9)	210	37 (17.6)	0.954

**Table 2:** Distribution of *S. aureus* among different samples isolated from cattle and buffalo farms through detection of the *nuc* gene.

Type of Samples	Cattle					Buffalo				
	Farm I		Farm II		*p* Value	Farm III		Farm IV		*p* Value
	*n*	Positive *n* (%)	*n*	Positive *n* (%)		*n*	Positive *n* (%)	*n*	Positive *n* (%)	
Rectal swabs	10	2 (20.0)	10	3 (30.0)	0.606	15	2 (13.3)	15	2 (13.3)	1.0
Udder swabs	10	3 (30.0)	10	2 (20.0)	0.606	15	2 (13.3)	15	3 (20.0)	0.624
Milk	10	1 (10.0)	10	2 (20.0)	0.531	15	0(0.0)	15	7 (40.0)	0.003 *
Subtotal	30	6 (20.0)	30	7 (23.3)	0.754	45	4 (8.9)	45	12 (26.8)	0.027 *
Bedding	10	2 (20.0)	10	2 (20.0)	1.0	15	1 (6.7)	15	3 (20.0)	0.598
Boot swabs	10	2 (20.0)	10	1 (10.0)	0.531	15	1 (6.7)	15	4 (26.7)	0.330
Feed	10	0 (0.0)	10	2 (20.0)	0.136	15	0 (0.0)	15	10 (66.7)	≤0.001 *
Water	10	0 (0.0)	10	3 (30.0)	0.06	15	0 (0.0)	15	2 (13.3)	0.483
Subtotal	40	4 (10.0)	40	8 (20.0)	0.21	60	2 (3.3)	60	19 (31.7)	≤0.001 *
Total	70	10 (14.3)	70	15 (21.4)	0.269	105	6 (5.7)	105	31 (29.5)	≤0.001 *

* Indicate significant differences (*p* < 0.05).

**Table 3:** Occurrence and distribution of enterotoxin and *mec*A gene among different sources.

Gene Function	Target Genes	Positive Samples *n*/Tested *n* (%)			
		Cattle	Buffalo	Karish Cheese	Total
Virulence genes	*sea*	10/25 (40.0)	6/37 (16.2)	2/5 (40.0)	18/67 (26.9)
	*seb*	0/25 (0.0)	0/37 (0.0)	0/5 (0.0)	0/67 (0.0)
	*sec*	2/25 (8.0)	0/37 (0.0)	1/5 (20.0)	3/67 (4.5)
	*sed*	0/25 (0.0)	1/37 (2.7)	1/5 (20.0)	2/67 (3.0)
	Total	12/25 (48.0)	7/37 (18.9)	4/5 (80.0)	23/67 (34.3)
Resistance gene	*mec*A	4/25 (16.0)	6/37 (16.2)	3/5 (60.0)	13/67 (19.4)

**Table 4:** Antimicrobial resistance profile of *S. aureus* isolates (*n* = 67) from animals, environment and Karish cheese.

Antimicrobial Agents	Concentration (µg)	Resistant *n* (%)
Clindamycin (CL)	10	67 (100.0)
Kanamycin (K)	30	65 (97.0)
Nalidixic acid (NA)	30	58 (86.6)
Cefotaxime (CF)	30	49 (73.1)
Sulphamethazole—trimethoprim (SXT)	25	44 (65.7)
Cefazolin (CZ)	30	36 (53.7)
Tetracycline (T)	30	31 (46.3)
Erythromycin (E)	15	25 (37.3)
Ampicillin (AM)	10	16 (23.9)
Gentamicin (G)	10	15 (22.3)
Imipenem (IPM)	10	9 (13.4)
Amikacin (AK)	30	4 (6.0)
Ciprofloxacin (CP)	5	3 (4.5)
Cefoxitin (FOX)	30	15 (22.3)

**Table 5:** Comparison of MAR index between different sample sources (livestock, dairy products and environment).

Cattle (*n* = 25)		Buffalo (*n* = 37)		*p* Value
Mean ± SD	Range	Mean ± SD	Range	
0.519 ± 0.235	0.14–1.0	0.382 ± 0.225	0.07–0.93	0.024 *
**Livestock (*n* = 29) and dairy products (*n* = 5) (*n* = 34)**		**Environment (*n* = 33)**		***p* Value**
0.519 ± 0.260	0.14–1.0	0.394 ± 0.211	0.07–0.79	0.035 *

* Indicate significant differences (*p* < 0.05).

**Table 6:** Mean ± SD, median, and range of MAR index for samples from different sources.

Sample	MAR Index			*p* Value
	Mean ± SD	Median	Range	
Rectal swabs	0.452 ± 0.319	0.357	0.14–1.0	0.027 *
Udder swabs	0.386 ± 0.241 ^ef^	0.286	0.14–0.79	
Milk	0.621 ± 0.161 ^adf^	0.607	0.36–0.93	
Karish	0.700 ± 0.216 ^bce^	0.786	0.36–0.93	
Bedding material	0.499 ± 0.174	0.535	0.29–0.71	
Boot swabs	0.321 ± 0.100 ^ab^	0.357	0.14–0.43	
Feed	0.363 ± 0.216 ^cd^	0.321	0.07–0.79	
Water	0.414 ± 0.347	0.286	0.07–0.79	

Similar superscripted letters denote significant differences between different samples. * Indicate significant differences (*p* < 0.05).

**Table 7:** Distribution of samples collected from different animal species, environments, milk, and Karish cheese.

Herd ID	Animal Species	Number of Animals/Herds	Samples Type and Number							
			Rectal Swabs	Udder Swabs	Milk	Bedding Materials	Boot Swabs	Feed	Water	Total
I	Cattle	37	10	10	10	10	10	10	10	70
II	Cattle	28	10	10	10	10	10	10	10	70
Subtotal		65	20	20	20	20	20	20	20	140
III	Buffaloes	38	15	15	15	15	15	15	15	105
IV	Buffaloes	39	15	15	15	15	15	15	15	105
Subtotal		77	30	30	30	30	30	30	30	210
Total		142	50	50	50	50	50	50	50	350
Karish cheese		30 samples of Karish cheese were collected from 10 different markets (three/market)								380

**Table 8:** Primer sequences of *nuc*, *mec*A, and enterotoxin gene for *S. aureus* detection used for PCR identification systems.

Target Gene	Oligonucleotide Sequence (5′→3′)	Product Size (bp)	References
*nuc* (F)	GCGATTGATGGTGATACGGTT	270	[55]
*nuc* (R)	AGCCAAGCCTTGACGAACTAAAGC		
*mec*A (F)	TAGAAATGACTGAAC GTCCG	533	[56]
*mec*A (R)	TTGCGATCA ATGTTACCGTAG		
*sea* (F)	TTGGAAACGGTTAAAACGAA	120	[47]
*sea* (R)	GAACCTTCCCATCAAAAACA		
*seb* (F)	TCGCATCAAACTGACAAACG	478	
*seb* (R)	GCGGTACTCTATAAGTGCC		
*sec* (F)	GACATAAAAGCTAGGAATTT	257	
*sec* (R)	AAATCGGATTAACATTATCC		
*sed* (F)	CTAGTTTGGTAATATCTCCT	317	
*sed* (R)	TAATGCTATATCTTATAGGG		

## Data Availability

Not applicable.
